# A dataset of synthetic face centered cubic 3D polycrystalline microstructures, grain-wise microstructural descriptors and grain averaged stress fields under uniaxial tensile deformation

**DOI:** 10.1016/j.dib.2018.06.072

**Published:** 2018-06-28

**Authors:** Ankita Mangal, Elizabeth A. Holm

**Affiliations:** Carnegie Mellon University, United States

## Abstract

This data article presents a data set comprised of 36 synthetic 3D equiaxed polycrystalline microstructures, the microstructural descriptors for each grain, and the stress and strain fields resulting from crystal plasticity simulations mimicking uniaxial tensile deformation to a total strain of 4%. This is related to the research article entitled “Applied Machine Learning to predict stress hotspots I: Face Centered Cubic Materials” (Mangal and Holm, 2018) [1]. The microstructures were created using an open source Dream.3D software tool, and the crystal plasticity simulations were carried out using the elasto-viscoplastic fast Fourier transform (EVPFFT) method. Six different kinds of FCC textures are represented with six stochastically different microstructures with varying texture intensity for each texture kind. This dataset is freely available in a Mendeley Data archive “A dataset of synthetic face centered cubic 3D polycrystalline microstructures, grain-wise microstructural descriptors and grain averaged stress fields under uniaxial tensile deformation” located at 〈http://dx.doi.org/10.17632/ss75fdg5dg.1〉 for any academic, educational, or research purposes.

## Specifications Table

**Table:** 

Subject area	*Materials Science*
More specific subject area	*Mechanical Metallurgy, Crystal Plasticity*
Type of data	*3D synthetic microstructures, Voxel-wise and grain-wise stress field, Taylor factors and microstructural descriptors, scripts*
How data was acquired	*3D microstructures were created using open source Dream.3D software*[4]*. Constitutive Modeling was done using EVPFFT*[2].
Data format	*Processed digital 3D microstructures in .HDF5 format, formatted scripts in .HTML format*
Experimental factors	*Microstructures are created at a resolution of 128 × 128 × 128 voxels, consisting of around 5000 grains each, with a lognormal grain size distribution and a mean grain size of 2.7 microns.*
	*The constitutive parameters represent a generic oxygen free high thermal conductivity(OFHC) copper alloy. The boundary conditions for EVPFFT crystal plasticity simulations correspond to uniaxial tension along Z, with an applied strain rate component along the tensile axis ε ˙*_*33*_*= 1 s*^*−1*^*. The simulation was carried out in 400 steps of 0.01%, up to a strain of 4%.*
Experimental features	*The Voce Hardening model was used to describe the strain hardening behavior during tensile deformation*
Data source location	*Pittsburgh, PA, USA*
Data accessibility	*Data are publicly available via Mendeley Data at*http://dx.doi.org/10.17632/ss75fdg5dg.1[3]
Related research article	*Dataset used in “Applied Machine Learning to predict stress hotspots I: Face Centered Cubic Materials” (Mangal and Holm, 2018)*[1]

## Value of the data

• This simulated dataset provides a statistically significant number of microstructures and stress fields along with grain-wise microstructural descriptors to analyze microstructure-property relationships in the context of material failure.
• The dataset can be used to develop and benchmark data driven analysis techniques for applications such as identifying grain size distribution from images and machine learning for understanding crystal plasticity behavior of FCC materials.
• The dataset can be used to pre-train machine learning models for transfer learning on experimentally obtained high energy electron diffraction (HEDM) measurements.
• The data may be compared with the tensile behavior of other face centered cubic materials.

## 1 Data

This data set consists of 36 synthetic 3D equiaxed polycrystalline microstructures with different cubic textures, associated microstructural descriptors, and the corresponding results from applying a uniaxial tensile stress through EVPFFT simulations.

## 2 Experimental design, materials, and methods

### 2.1 Synthetic microstructure generation pipeline

A dataset of synthetic microstructure images is built to be used as input to a parallelized elasto-viscoplastic Fast Fourier Transform (EVPFFT) crystal plasticity formulation [2]. Dream.3D (Digital Representation Environment for Analyzing Microstructure) is an open source software package [4], [5] used to create synthetic three-dimensional polycrystalline microstructures. Dream.3D uses objects (ellipsoids, octahedrons, cylinders) to represent grains. These shapes are evolved to fill a predefined cubic volume to match the statistics specified above. The microstructure is discretized on a 128 × 128 × 128 grid. The first step in the pipeline is to define microstructural statistics such as grain size distribution and texture (Euler angle distribution). This is done using the Stats Generator filter inside Dream.3D. The following statistics can be generated in this program:

• Phase Types: primary, precipitate, matrix, transformation.
• Relative volume fraction of phases.
• Crystal structure of each phase.
• Grain size distribution (parameters of the lognormal distribution).
• Grain shape: aspect ratios of grains defining them as equiaxed or rolled.
• Neighbor distributions: parameters of the lognormal distribution.
• Texture: The crystallographic orientation in the form of orientation distribution function (ODF) or misorientation distribution function (MDF).

**Fig. 1:**
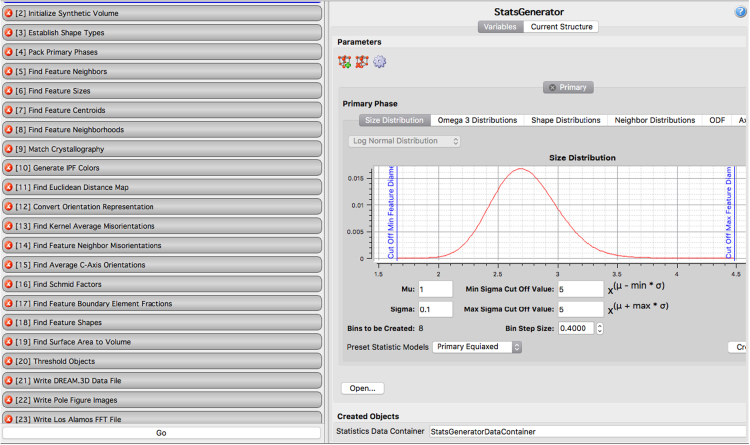
Screenshot of DREAM.3D showing the synthetic microstructure construction pipeline.

Table 1 shows the DREAM.3D parameters used to generate the microstructures included in this data set, and the DREAM.3D workflow used is shown in Fig. 1. The representative Euler angles used to generate the texture distribution in the Stats Generator filter for the 6 microstructures are listed in Table 2. The texture intensity was varied to get 6 different instantiations within each representative texture. An example microstructure, showing the equiaxed grain geometry typical of all the samples, is shown in Fig. 3, and the six crystallographic textures are represented as pole figures in Fig. 4. Overall, 36 3D microstructures are included in the data set: Six each of the six textures.

**Table 1:** Dream.3D parameters.

**Phase type:**	Primary	**Grain shape**	Equiaxed
**Mean Grain Size (*μ*)**	2.3 microns	**Standard deviation (*σ*)**	0.4 microns
**Bin Step Size**	10	**Bins created**	4
**Max Grain Size cutoff**	*μ* + 3*σ*	**Min Grain Size cutoff**	*μ* − 4*σ*

**Table 2:** Bunge Euler angles (*ϕ*1, *θ*, *ϕ*2) used to generate the 6 representative textures.

	***ϕ*1**	**θ**	***ϕ*2**		***ϕ*1**	***θ***	***ϕ*2**
**Micro1**	n/a	n/a	n/a	**Micro4**	90	35	45
**Micro2**	35	45	0	**Micro5**	55	30	65
**Micro3**	59	37	63	**Micro6**	45	35	65

**Fig. 2:**
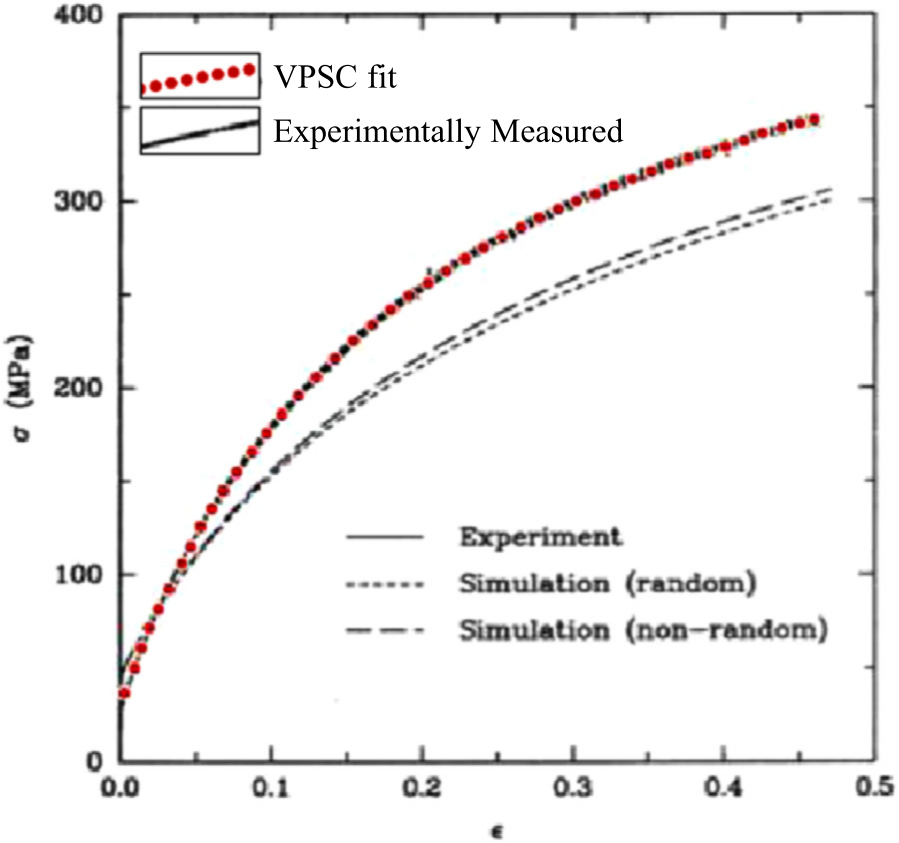
VPSC simulation fit to the experimentally observed stress–strain curve for OFHC Copper.

**Fig. 3:**
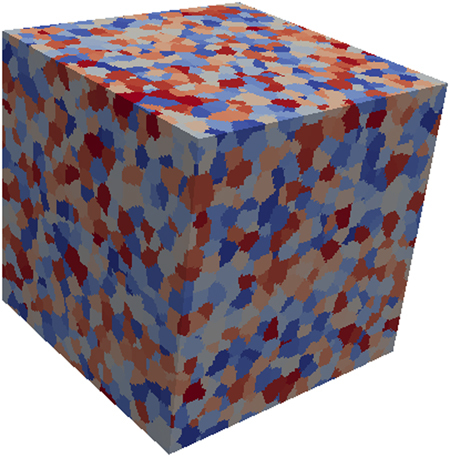
A sample polycrystalline microstructure with equiaxed grains, generated using Dream.3D.

**Fig. 4:**
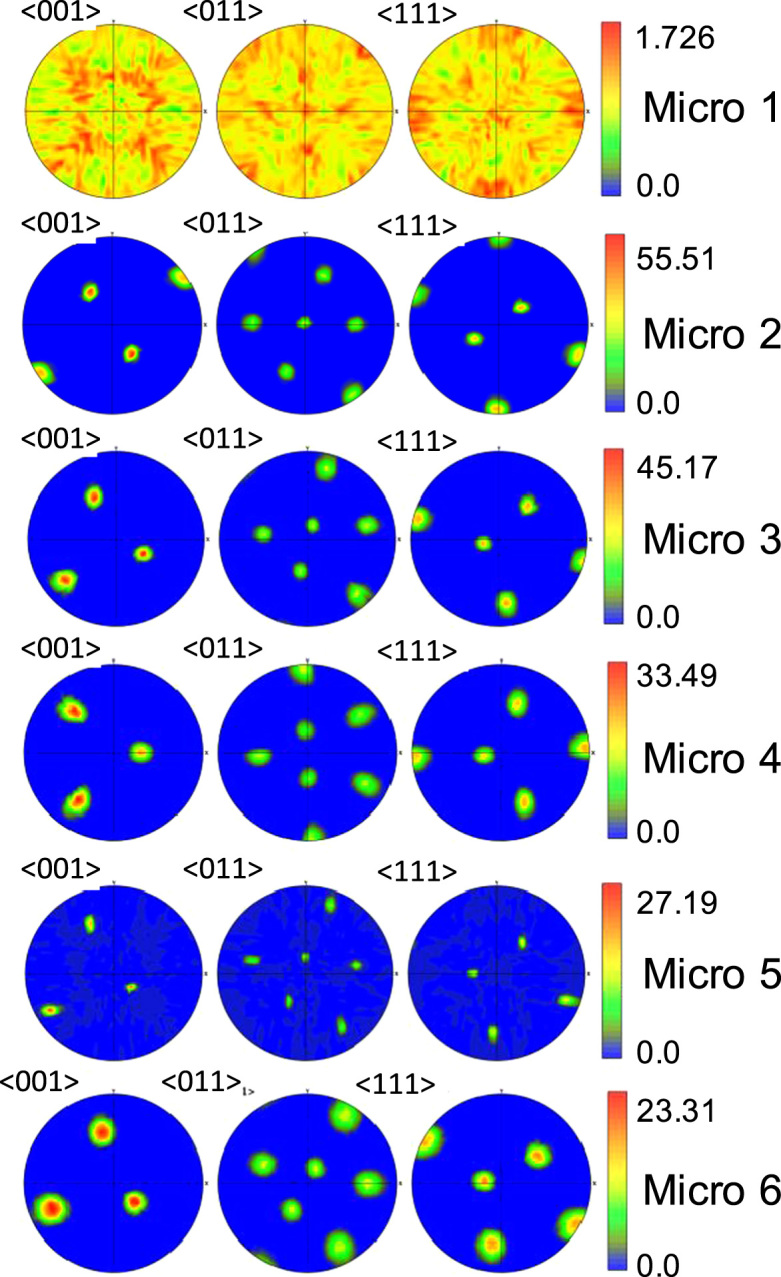
Representative pole figures for 6 different kinds of FCC textures chosen.

### 2.2 Micromechanical modeling

We use an elasto-viscoplastic model based on fast Fourier transforms (EVPFFT) [2], [6], [7] to calculate the local stress and strain fields that develop in these synthetic microstructures when they are subjected to a uniaxial tensile deformation. A 4% strain was chosen so that materials would transition from elastic to plastic deformation. The constitutive model parameters for FCC materials represent oxygen free high thermal conductivity (OFHC) copper with single crystal elastic constants given in Table 3. FCC materials deform plastically by slip on twelve {111} < 110 > slip systems. To obtain the actual values of the critically resolved shear stress (CRSS) and the Voce hardening parameters, the Voce model was fit to an experimentally measured stress–strain curve for uniaxial tension in OFHC copper [8] using the viscoplastic single crystal (VPSC) formulation similar to [9]. The results of the fitting are shown in Fig. 2. The Voce hardening parameters for this case are shown in Table 4. The boundary conditions correspond to uniaxial tension along *Z*, with an applied strain rate component along the tensile axis *ε* ˙_33_ = 1 s^−1^. The EVPFFT simulation was carried out in 400 strain steps of 0.01%, up to a strain of 4%. The microstructure and EVPFFT results files are contained in the FCC_voxelwise folder; Table 7 describes the data contained in these files. The EVPFFT code package can be obtained by contacting the Richard P. Feynman Center for Innovation at Los Alamos National Laboratory [10].

**Table 3:** Single crystal elastic stiffness constants (in GPa).

**Material**	***C***_**11**_	***C***_**12**_	***C***_**44**_
Copper	168.4	121.4	75.4

**Table 4:** Voce hardening parameters imitating copper.

**CRSS ratio**	$\tau_{0}^{s}$**(MPa)**	$\tau_{1}^{s}$**(MPa)**	$\theta_{0}^{s}$	$\theta_{1}^{s}$
1:1:1	7.43	102.79	356.44	13.01

## 3 Microstructural descriptors

For enabling machine learning, we constructed a set of crystallography and geometry based microstructural descriptors. Dream.3D filters provide a convenient way to calculate a number of microstructural descriptors. Table 5 describes how the crystallographic descriptors were computed. Table 6 describes how the geometry based descriptors were computed. The processed grain-wise descriptors and grain-wise EVPFFT simulation results are contained in the FCC_grainwise folder; Table 7 describes the data contained in these files.

**Table 5:** Computing crystallographic descriptors.

**Feature type**	**Measured by:**
Distance from Inverse pole figure corners:	This distance measures the orientation of the sample direction with respect to the [001, 101] and [111] crystal directions. This is calculated from grain Euler angles using a custom function included in the script.
Average Misorientation	A Dream.3D filter “Find Feature Neighbor Misorientations” is used to calculate the list of misorientations between a grain and its nearest neighbors. This list is averaged to get the metric.
Slip Transmission Metrics	A Dream.3D filter “Find Neighbor Slip Transmission Metrics” is used to calculate the geometric compatibility factor “m prime” measuring the ease of slip transmission [11] across the grain boundaries.
Schmid Factor	A Dream.3D filter “Find Schmid Factors” is used to calculate the Schmid factor from the average orientation of a grain given a loading direction.

**Table 6:** Computing geometry descriptors.

**Feature type**	**Measured by:**
Grain Size measurements	Different metrics of grain size such as equivalent spherical diameter, number of voxels in a grain and number of neighbors are calculated using the following Dream.3D filters: “Find Feature Sizes” and “Find Feature Neighbors”
Grain shape parameters	The grain shapes are measured using their aspect ratios and surface area to volume ratio. These properties are calculated using to the Dream.3D filters “Find Feature Shapes” and “Find Surface Area to Volume”
Shape averaged distance from special points	The Dream.3D filter “Find Euclidean Distance Map” computes the distance of each voxel from its nearest grain boundary, triple junction and quadruple point. The voxels belonging to each grain are averaged to get a shape averaged distance from the special points (grain boundary, triple junction and quadruple point).
Grain fraction on the periodic boundary	A Dream.3D filter “Find Feature Boundary Element Fractions” is used to calculate the fraction of voxels in each grain that lie at 3D image boundary

**Table 7:** Dataset file structure.

**Data element**	**Description**	
**Directory**		
**FCC_voxelwise**	Contains the EVPFFT plasticity simulation inputs and results, comprised of a voxel-wise representation of microstructure, stress, and strain fields. Files represent 6 kind of textures (X), with 6 stochastic instantiations per texture (Y), with filenames parsed as microX_Y_voxel.hdf5.	
**Files**		
micro1_1_voxel.hdf5	**Dataset keys:**	**Remarks:**
micro1_2_voxel.hdf5		
.	EulerAngles	Voxel-wise Euler Angles (Kocks convention)
.	ngr	Voxel-wise Grain IDs
.		
micro1_6_voxel.hdf5	DistanceFrom	Voxel-wise distance from grain boundaries, triple junctions and quadruple points
.		
.		
	Taylor	Taylor factor calculated by EVPFFT
.		
micro6_6_voxel.hdf5		
	XYZ	Location of the voxel in 3D volume of size 128 × 128 × 128
	strains	Voxel-wise components of the strain tensor:
		[׳e11׳, ׳e22׳, ׳e33׳, ׳e23׳, ׳e13׳, ׳e12׳]
	stresses	Voxel-wise components of the stress tensor:
		[׳str11׳, ׳str22׳, ׳str33׳, ׳str23׳, ׳str13׳, ׳str12׳]
**Directory**		
**FCC_grainwise**	Contains the processed microstructural descriptor data including the grain-wise representation of microstructure, microstructural features, equivalent Von Mises stress, and strain field. Data are aggregated from the 6 instantiations per texture (Y), with filenames parsed as microY_all_grainwise.csv.	
**Files**		
micro1_all_grainwise.csv	**Dataset keys:**	**Remarks:**
.		
	[‘fileID’]	Identifier within each texture kind
.		
	[‘ngr’]	Grain ID
micro2_all_grainwise.csv		
.	[׳001_IPF_0׳, ׳001_IPF_1׳, ׳001_IPF_2׳]	Distance from 3 corners of the 001 inverse pole figure as described in Mangal and Holm [1].
.		
micro6_all_grainwise.csv	[׳100_IPF_0׳, ׳100_IPF_1׳, ׳100_IPF_2׳]	Distance from 3 corners of the 100 inverse pole figure as described in Mangal and Holm [1].
	[׳111_IPF_0׳, ׳111_IPF_1׳, ׳111_IPF_2׳]	Distance from 3 corners of the 111 inverse pole figure as described in Mangal and Holm [1].
	[׳AspectRatios_0׳, ׳AspectRatios_1׳, ׳Omega3s׳]	Aspect ratios of the grain according to the Dream.3D filter
	[׳EqVonMisesStress׳]	Equivalent Von Mises Stress in the grain
	[‘hotspot’]	True if the grain is a stress hotspot
	[׳euler_1׳, ׳euler_2׳, ׳euler_3׳]	Grain-wise Euler Angles
	[׳GBEuc׳, ׳TJEuc׳, ׳QPEuc׳]	Grain-averaged distance from grain boundaries, triple junctions and quadruple points
	[‘Taylor’]	Taylor factor calculated by EVPFFT
	[‘Schmid’]	FCC Schmid factor
	[׳F1List׳, ׳F1sptList׳, ׳F7List׳,׳mPrimeList׳]	Slip transmission metrics
	[׳NeighborList׳, ׳MisorientationList׳, ׳AvgMisorientations׳]	List of grain neighbors, grain neighbor misorientations and the average misorientation per grain calculated using Dream.3D filters
	[׳EquivalentDiameters׳, ׳FeatureVolumes׳, ׳NumCells׳, ׳Neighborhoods׳, ׳NumNeighbors׳, ׳SurfaceAreaVolumeRatio׳, ׳SharedSurfaceAreaList׳ ]	Different measures of grain size calculated using Dream.3D filters
	[׳SurfaceFeatures׳, ׳FeatureBoundaryElementFrac׳]	Metrics describing if grain lies on the 3D image boundary calculated using Dream.3D filters
**Directory**		
**Images**	Contains the processed images representing pole figures and simulated stress strain curves for each microstructure	
**Subdirectory**	PoleFigures	FlowCurves
**Directory**		
**Code**	Contains the open source jupyter notebook in .HTML format describing the two datasets and how to read them in Python	
